# Cutaneous Leukocytoclastic Vasculitis Induction Following ChAdOx1 nCoV-19 Vaccine

**DOI:** 10.7759/cureus.19005

**Published:** 2021-10-24

**Authors:** Shaghayegh Shahrigharahkoshan, Louis-Philippe Gagnon, Steve Mathieu

**Affiliations:** 1 Dermatology, Laval University, Quebec City, CAN; 2 Pathology, Laval University, Quebec City, CAN

**Keywords:** immune response, vaccine, covid-19, chadox1 ncov-19, cutaneous vasculitis

## Abstract

The coronavirus disease 2019 (COVID-19) pandemic is an unprecedented event, and in order to control its spread and minimize its damages, all efforts are immediately mobilized. Mass vaccination is considered a promising solution to combat this universal issue. However, given the urgent need for vaccine production, some of the side effects may not have been presented during trials and will only appear during the mass vaccination. Limited vasculitis cases have been reported so far following vaccination against COVID-19. We present a case of cutaneous leukocytoclastic vasculitis (LCV) induced following the first dose of the ChAdOx1 nCoV-19 vaccine in an otherwise healthy individual.

## Introduction

The cutaneous reactions after vaccination against coronavirus disease 2019 (COVID-19) are not rare, and a limited number of vasculitides have been reported so far. The vaccine has been proven to be a safe and effective intervention through numerous clinical trials, and immunization is considered the most promising approach for the protection against coronavirus. However, the urgent need for its efficient production limited the vaccine trials to account for the long-term events before mass vaccination [1]. To date, many vaccines against severe acute respiratory syndrome coronavirus 2 (SARS-CoV-2) have been developed. The ChAdOx1 nCoV-19 vaccine is a viral vector vaccine consisting of a replication-deficient adenovirus vector ChAdOx1 which contains the structural surface glycoprotein (spike protein nCoV-19) of SARS-CoV-2. Large-scale clinical trials have shown its safety and efficacy, and it has been distributed worldwide since its approval [2].

Vasculitis denotes the inflammation of the blood vessel walls by leukocytes. The most frequent variant of vasculitis in the skin is leukocytoclastic vasculitis (LCV), causing the acute necrotizing inflammation of small vessels and post-capillary venules with neutrophilic infiltration, leukocytoclasia, fibrin deposition, and hemorrhage. The triggering factor of LCV usually remains unknown, but it can be associated with drug reactions, chemicals, microorganisms, or as a consequence of other diseases [3]. Herein we present a case of cutaneous LCV, which was triggered 10 days after receiving the first dose of the ChAdOx1 nCoV-19 vaccine.

## Case presentation

**Figure 1:**
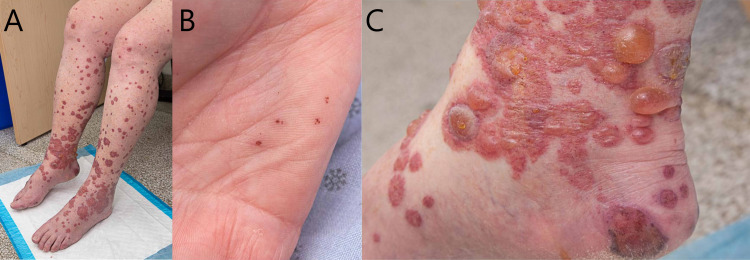
Purpuric manifestation of cutaneous leukocytoclastic vasculitis on the (A) legs, (B) palmar surface of the hand, and (C) internal malleolus

An otherwise healthy 77-year-old woman was referred to our dermatology center, presenting progressive abrupt maculopapular eruptions starting with petechia at the lower extremities progressing in a 24-hour interval towards the shins and calves. She had no fever, no respiratory, gastrointestinal, or urinary symptoms, nor did she have a relevant medical history. At the physical examination, multiple palpable indurated purpuric papules along with erythematous plaques and bullae, some of which were coalescing, were present on the lower extremities (Figure 1A) as well as on the palmar face of the hands (Figure 1B). Several purpuric lesions were also noticed on the soft palate and the tongue, sparing face, eyes, and trunk areas, along with numerous pseudo-vesicles around the ankles (Figure 1C).

**Figure 2:**
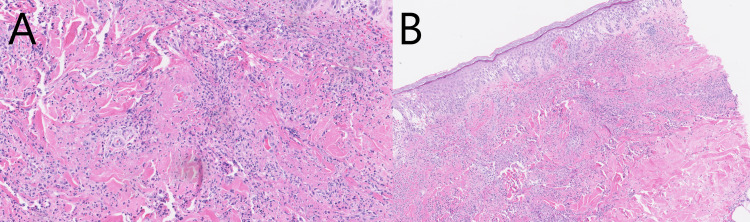
Hematoxylin and eosin (H&E) stained histology sections showing LCV changes in the dermis at (A) 100x magnification and (B) 40x magnification

Punch biopsy of the calf confirmed the diagnosis of cutaneous LCV (Figure 2).

No microorganisms were observed using the periodic acid-Schiff diastase (PASD), Gram, and Fite stains. No vascular deposit for IgG, IgM, IgA, and C3 was seen in the direct immunofluorescence study.

**Figure 3:**
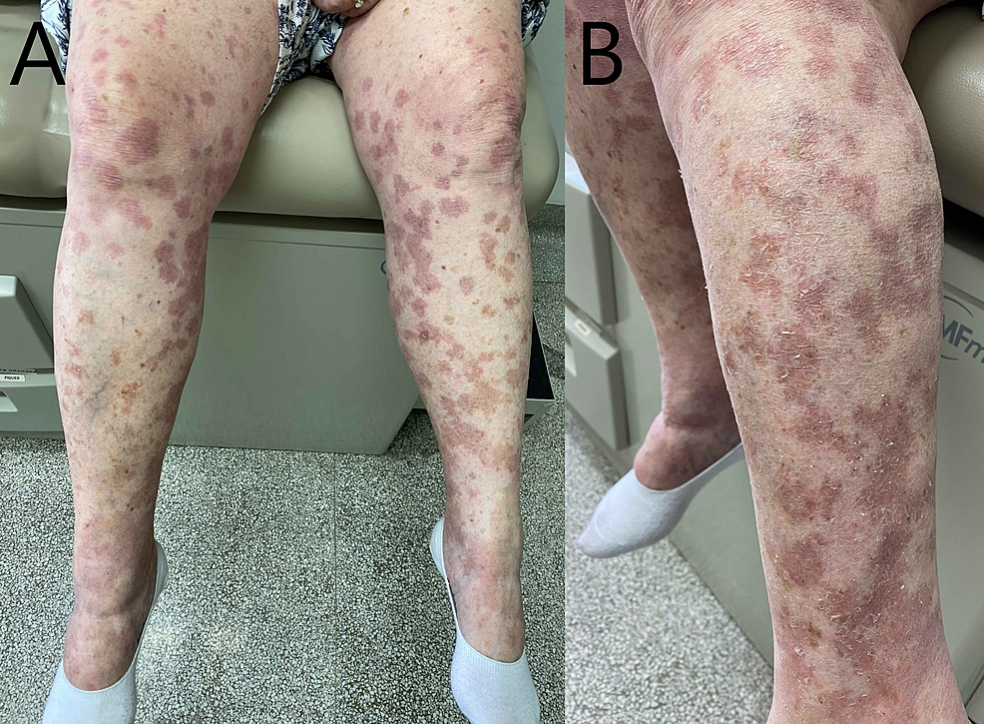
The lesions are healing after dapsone and corticosteroid therapy on (A) both legs, and (B) the pseudo-vesicles were cleared

Initial laboratory tests were normal except for the slight elevation of erythrocyte sedimentation rate and C-reactive protein. The blood, urine, and superficial pus cultures were negative. The dosage of antistreptolysin-O, antineutrophil cytoplasmic antibodies (ANCA), glucose-6-phosphate dehydrogenase, reticulocytes, cryoglobulin, and serum proteins were non-conclusive. Prednisolone 50 mg per day was started for her immediately, which was tapered by 5 mg every three days. Additionally, dapsone 50 mg daily was prescribed for 60 days. She showed improvement, and vasculitis was resolved following treatment, but limited residual blanching macules and patches were still present on the previously affected sites (Figure 3). We recommended a different vaccine for the second dose. No similar reaction was observed following receiving a different vaccine at her five-month follow-up.

## Discussion

Herd immunization is considered a promising solution to manage the COVID-19 pandemic. Similar to other vaccines such as influenza, hepatitis B, human papillomavirus, meningococcal vaccines [4], cutaneous side effects to the COVID-19 vaccine have been reported. Given the urgent need for vaccine fabrication, some of the long-term side effects may not have been presented during clinical trials and will only surface during the mass vaccination. The most common side effects include local reactions such as erythema, swelling, and urticarial eruptions [5,6]. Limited vasculitis cases following any COVID-19 vaccine have been reported so far. An international registry of 414 cutaneous reactions following Pfizer-BioNTech, and Moderna vaccines reported three vasculitides [5]. LCV is a rare vaccine side effect; however, recent case reports presented the flare of LCV, antineutrophil cytoplasmic antibody vasculitis, and varicella-zoster-related vasculitis following Pfizer-BioNTech vaccine [7-9] and cutaneous vasculitis following inactivated whole virion vaccine [10]. To the best of our knowledge, no LCV case following the ChAdOx1 nCoV-19 vaccine has been reported so far.

The induction of cutaneous vasculitis following infection by SARS-CoV-2 is widely established [11,12]. In the skin biopsies of SARS-CoV-2 induced cutaneous LCV, cytoplasmic granular positivity for SARS-CoV-2 spike protein was observed, which is also the end product of ChAdOx1 nCov-19 vaccine [13]. This protein will activate the host immune system and stimulate antibody secretion. In other words, the vaccine end product serves as a triggering factor for the secretion of the circulating antibodies and the subsequent antigen-antibody complex formation, which will be eventually deposited within the post-capillary venules and trigger an inflammatory cascade leading to vascular endothelium damage and vasculitis [11]. In addition, it is shown that some comorbid conditions such as advanced age, diabetes mellitus, hypertension, and obesity are associated with endothelial damage rendering the occurrence of vasculitis after COVID-19 more probable [11]. Other than advanced age, no other triggering factors could explain this association in our patient. Finally, It has also been postulated that the resemblance of the vaccine-induced spike proteins with human components may result in the production of pathological autoantibodies and vaccine-induced autoimmunity through molecular mimicry [14].

## Conclusions

Vasculitides are a group of diseases that may have serious consequences for the patient if not managed appropriately. By being aware of this possible complication of the ChAdOx1 nCoV-19 vaccine, healthcare professionals would be better prepared and equipped to prevent its grave consequences, particularly when other vital organs are involved. Finally, by presenting this case, we do not necessarily draw a causal relationship between vasculitis and the ChAdOx1 nCoV-19 vaccine, but given the possible morbid or even fatal course of vasculitis, this relationship warrants careful investigations in the clinical trials.
